# Cognitive performance, dizziness and tinnitus among adults with unilateral audiovestibular disorders

**DOI:** 10.3389/fneur.2026.1911120

**Published:** 2026-08-17

**Authors:** Maura Cosetti, Carly Fiest, Liraz Arie, Jennifer Kelly, Anat Vilnai Lubetzky

**Affiliations:** 1 IDepartment of Otolaryngology-Head and Neck Surgery, Icahn School of Medicine at Mount Sinai, New York, NY, United States; 2 Ear Institute, New York Eye and Ear Infirmary of Mount Sinai, New York, NY, United States; 3 Department of Physical Therapy, Cognitive-Motor Behavior Laboratory, MGH Institute of Health Professions, Boston, MA, United States; 4 Department of Physical Therapy, Steinhardt School of Culture, Education and Human Development, New York University, New York, NY, United States

**Keywords:** cognition, dizziness, peripheral vestibulopathy, tinnitus, unilateral hearing loss

## Abstract

**Objective:**

To investigate cognitive performance in patients with unilateral audiovestibular disorders, specifically hearing loss (UHL), vestibular hypofunction (UVH), and Meniere’s Disease (MD) using a visually administered, computerized domain-specific cognitive battery.

**Materials/methods:**

58 adults: 28 controls (mean age 59 years, range 41–78) and 30 patients (10 UHL [4Frequency Pure Tone Average <25 dB in better ear; >40 poor ear] mean age 55, range 25–76; 10 unilateral MD, mean age 53, 32–78; 10 UVH, mean age 59, 24–82) completed the cognitive test battery and the Dizziness Handicap Inventory (DHI), Tinnitus Handicap Inventory (THI), Speech, Spatial and Qualities of Hearing scale (SSQ12), Activities Specific Balance Confidence Scale (ABC). Age-standardized scores as well as proportion of patients below average were compared between groups and correlations between cognitive performance and symptom severity were explored in clinical groups.

**Results:**

Only 3 domains differed significantly between groups (verbal memory [*H*(3) = 9.28, *p* = 0.026], working memory [*H*(3) = 7.77, *p* = 0.05] and sustained attention [*H*(3) = 9.35, *p* = 0.03]); and only 2 differences remained significant after Bonferroni correction for multiple comparisons: MD scored significantly lower than controls in verbal memory ( $p_{\text{Bonf}}=0.0174$ ) and patients with UHL group scored significantly lower than controls in sustained attention ( $p_{\text{Bonf}}=0.0391$ ). Proportion of individuals in each group performing “below average” (standardized score < 90) on each domain trended toward lower performance on the domains of verbal memory for UHL and MD groups, executive function for MD, and sustained attention for those with UHL or UVL. The latter 2 maintained significance after Bonferroni correction. Overall, less symptom severity (lower THI and DHI scores) correlated with better cognitive function, especially in patients with MD.

**Conclusion:**

Cognitive effects of unilateral audiovestibular disorders represent an important knowledge gap in our understanding of these disorders. In this exploratory pilot study, UHL, MD and UVH demonstrated differences in cognitive performance compared to controls along domains related to memory and sustained attention that may reflect challenges unique to hearing loss, vestibular loss or a combination. Severity of disease related symptoms, specifically dizziness and tinnitus, may also relate to cognitive performance, especially in patients with MD. Further study exploring the cognitive burden of unilateral audiovestibular disease is warranted.

## Introduction

The relationship between bilateral hearing loss and dementia has been widely investigated, with a large body of literature confirming bilateral hearing loss as an independent risk factor for cognitive decline (1–3). The 2017, 2020 and 2024 Lancet Commissions on Dementia Prevention, Intervention, and Care identified untreated midlife hearing loss as the largest potentially modifiable risk factor for dementia, accounting for 7–8% of cases (1, 4, 5). More recently, intervention studies have demonstrated the potential for treatment of hearing loss, including with hearing aids or cochlear implants, to slow cognitive decline (6–8).

As stated, these studies have examined populations with bilateral hearing loss, or have categorized the target populations based on their better hearing ear, thereby obscuring presence of a unilateral loss. Auditory effects of unilateral sensorineural hearing loss (specifically profound loss, such as single-sided deafness, SSD) are well-characterized and include impaired speech perception- especially in background noise- and absent (or significantly impaired) spatial hearing (9, 10). Beyond audition, affected adults have reduced quality of life (QoL,) increased fatigue, and many experience considerable tinnitus (11, 12). Overall, few studies have examined the relationship between cognitive function and unilateral hearing loss (UHL) in adults, with most focusing on the increase in listening effort required to process auditory stimuli with an impaired ear (13). It is conceptualized that the brain needs to allocate more resources to compensate for poor hearing, thereby increasing cognitive load and reducing other cognitive functions such as memory and attention (13). Initially postulated as an underlying mechanism for the relationship between bilateral hearing loss and cognitive decline, this theory has not been thoroughly investigated in UHL. Overall, the impact of UHL on non-auditory domains of cognition or on cognitive decline is currently unknown (14).

Clinicians treating dizzy patients of various etiologies often encounter complaints related to cognitive function, such as brain fog, difficulty concentrating, confusion and forgetfulness, and frequently transcend or continue between episodes (15–17). Vestibular disorders comprise a diverse group of pathologies that include unilateral or bilateral peripheral disorders, central etiologies, and those with episodic or non-episodic symptoms. Although studies on vestibular dysfunction and cognition are comparatively fewer than those on hearing loss, some cross-sectional, epidemiologic studies have found increased rates of cognitive impairment among adults with various types of vestibulopathies (18, 19). Interestingly, clinical studies examining particular vestibular diagnoses and cognitive testing have shown mixed results, suggesting that the relationship is complex and may be disease-specific (18). For example, patients with bilateral vestibular dysfunction and those with chronic vestibulopathy (such as Persistent Postural-Perceptual Dizziness [PPPD]) exhibited worse cognitive function than those with acute vestibular syndromes or otolithic pathology such as Benign Paroxysmal Positional Vertigo (BPPV) (18). The cognitive test batteries used varied widely between the studies, with the majority focused on the domain of visuospatial memory and orientation (19, 20). In contrast, fewer studies have examined the general effects of vestibular dysfunction on non-spatial cognitive performance. Additionally, among the > 45 included studies in the recent systematic review, few noted the presence of concomitant hearing loss or incorporated this potential confounder it into their analyses (18).

Both hearing loss and vertigo are key features of Meniere’s disease (MD) a chronic inner-ear disorder defined by episodic vertigo, fluctuating sensorineural hearing loss, tinnitus, aural pressure and progressive loss of audiovestibular function in the affected ear (21). The few studies examining cognitive function in MD have demonstrated conflicting results, and without clear understanding of whether cognition may be influenced by symptom and/or disease severity (22–24).

The overall purpose of the present pilot study was to investigate domain-specific cognitive performance in unilateral audiovestibular disorders characterized by hearing loss, vestibular dysfunction or both, and the relationship to symptoms. The first aim compared performance between healthy controls and patients with unilateral hearing loss (UHL), unilateral vestibular hypofunction (UVH), and unilateral Meniere’s Disease (MD) across a broad, non-auditory cognitive test battery (specifically the Central Nerves System Vital Sign; CNSVS.) In this aim, the primary exploratory analysis utilized continuous age-adjusted scores across all domains without prespecified domains of interest. Secondary descriptive analysis examined those participants whose performance was classified as below average (as defined by the CNSVS; additional detail in methods).

The second aim explored the relationship between cognitive performance and disease symptoms as measured by validated Patient-Reported Outcome Measures (PROMs). Dizziness, tinnitus, hearing and balance confidence have been related to cognitive function (22, 25–28). This aim explored how domain-specific cognitive function relates to patient reported measures of dizziness, tinnitus, hearing and balance confidence across 3 clinical groups.

## Methods

### Participants

Controls without known hearing or vestibular loss were recruited through the community. Patients with MD, UHL loss and UVH were recruited from the New York Eye & Ear Institute of Mount Sinai (NYEEIMS). Comprehensive audiometry including ear-specific air and bone conduction thresholds was performed in a soundproof booth.

UHL: Individuals with UHL were included if they had unilateral sensorineural hearing loss [i.e., 4 frequency pure-tone average (4F PTA > 40 dB)], normal hearing in one ear (i.e., 4F PTA < 25 dB) for a minimum of 1 year.

MD: Patients with episodic vertigo and unilateral hearing loss were included if they met clinical criteria for definitive unilateral MD, as defined by according to the Bárány Society and American Academy of Otolaryngology-Head and Neck Surgery diagnostic criteria (29).

UVH: Defined as >25% weakness on bithermal caloric testing during videonystagmography and/or positive bedside head impulse test consistent with unilateral peripheral hypofunction and normal hearing. Individuals with known Persistent Postural-Perceptual Dizziness (PPPD) were excluded.

Recruitment of patients and all testing was performed at an urban, tertiary referral outpatient clinic. All testing occurring on the same day whenever possible, or on two separate occasions per participant preference. The complete test battery took an average of 1 h. Participants were excluded for conductive hearing loss or air bone gap, bilateral vestibular disease, visual impairment above 20/63 on the Early Treatment Diabetic Retinopathy Study Acuity Test that cannot be corrected with lenses, recent sudden hearing loss, pregnancy, neurological conditions affecting cognition, and inability to read and provide informed consent in English. Additionally, patients with active BPPV, definite vestibular migraine (VM) (30) or had known involvement of the central nervous system that may be responsible for vestibular or auditory symptoms (i.e., stroke, multiple sclerosis, vestibular schwannoma, tumors, etc.) were also excluded.

The study was conducted under the approval of the Icahn School of Medicine at Mount Sinai’s Institutional Review Board STUDY-21-01026 the New York University Institutional Review Board (IRB-FY2021-5400.) All participants provided a written informed consent prior to commencing study procedures.

### Procedures

Participants completed a ~30 min visual-based computerized cognitive battery (Central Nerves System Vital Sign; CNSVS). The CNSVS is a widely used battery with extensive age-based norms and was selected for its ability to capture data on working memory, sustained attention, complex reaction time and executive function in a visual battery (to eliminate the confounders of hearing loss) (31). Domains of the CNSVS computerized cognitive battery is described in detail in Table 1. CNSVS standard scores were calculated from the raw scores according to age-matched, healthy normative data and had a mean of 100 (standard deviation [SD] 15). Higher scores indicate better performance, except for complex reaction time, where lower scores reflected better performance. If any invalid test results were identified, the test was repeated. The severity classification grade for the standardized score is above >110, average 90–110, low average 80–90, low 70–79 and very low <70.

**Table 1:** Description of the CNSVS computerized cognitive battery cognitive domains.

Cognitive domain	Description	Tests	Formula
Composite memory	Measure: How well a subject can recognize, remember, and retrieve words and geometric figures. Relevance: Remembering a scheduled test, recalling an appointment, taking medications, and attending class.	‘Verbal Memory’ = measures immediate and delayed recognition memory for words. ‘Visual Memory’ = measures immediate and delayed recognition memory for abstract figures and shapes.	‘Verbal Memory’ score + ‘Visual Memory’ score
Reaction Time	Measure: How quickly the subject can react, in msec, to a simple and increasingly complex direction set. Relevance: Driving a car, attending to conversation, tracking and responding to a set of simple instructions, taking longer to decide what response to make.	‘Stroop test’ Part 2 = response in msec to Part 2 in which words match the color. ‘Stroop test’ Part 3 = response in msec to Part 3 in words do not match the color.	(‘Stroop Test’ Part 2 msec + ‘Stroop Test’ Part 3 msec)/2
Cognitive Flexibility	Measure: How well a subject can adapt to rapidly changing and increasingly complex set of directions and/or to manipulate the information. Relevance: Reasoning, switching tasks, decision-making, impulse control, strategy formation, attending to conversation.	‘Shifting Attention’ = responds to a set of rules that change every half a second. ‘Stroop test’ Part 3 = words do not match the color.	‘Shifting Attention’ correct responses - ‘Shifting Attention’ Errors - ‘Stroop test’ Part 3 Errors
Executive Function	Measure: How well a subject recognizes rules, categories, and manages or navigates rapid decision making. Relevance: Ability to sequence tasks and manage multiple tasks simultaneously as well as tracking and responding to a set of instructions.	‘Shifting Attention’ = responds to a set of rules that change every half a second.	‘Shifting Attention’ Correct Responses – ‘Shifting Attention’ Errors
Working Memory	Measure: How well a subject can perceive and attend to symbols using short-term memory processes. Relevance: Ability to carry out short-term memory tasks that support decision making, problem solving, planning, and execution. Enables “right-now” responses.	‘4PCPT’ Part 4 = quickly respond if the current figure matched the one presented two figures earlier.	‘4PCPT’ Part 4 Correct Responses-‘4PCPT’ Part 4 Errors
Sustained Attention	Measure: How well a subject can direct and focus cognitive activity on specific stimuli. Relevance: How well a subject can focus and complete task or activity, sequence action, and focus during complex thought.	‘4PCPT’ Part 2 = quickly respond to one simple stimulus. ‘4PCPT’ Part 3 = quickly respond if a figure matches the one presented one figure earlier. ‘4PCPT’ Part 4 = quickly respond if the current figure matched the one presented two figures earlier.	(4PCPT Part 2 Correct Responses + 4PCPT Part 3 Correct Responses + 4PCPT Part 4 Correct Responses) – (4PCPT Part 2 Errors + 4PCPT Part 3 Errors + 4PCPT Part 4 Errors)

4PCPT, 4-Part Continuous Performance; msec, milliseconds.

We collected the following patient-reported outcomes: the Dizziness Handicap Inventory (32) (DHI), Activities Specific Balance Confidence Scale (33) (ABC), the Speech, Spatial, and Qualities of Hearing Scale (34) (SSQ-12), and the Tinnitus Handicap Inventory (35) (THI). The DHI is a well-established, 25-item questionnaire used to quantify a patient’s self-perceived handicap because of dizziness (32). The ABC assesses perceived % balance confidence when completing various functional mobility tasks, with 100% being complete confidence in balance (33). The SSQ12 measures self-reported auditory disability across three domains (speech hearing, spatial hearing, and qualities of hearing), with a score of 10 being perfect hearing and lower scores reflecting impairment (34). Finally, THI is a 25-item validated metric for assessing tinnitus severity, with higher scores representing greater handicap (i.e., worse severity of tinnitus) (35).

### Statistical analysis

We used descriptive statistics for all variables. Aim 1 compared continuous variables (8 age-adjusted standardized scores) using the independent samples Kruskal–Wallis test followed by Mann–Whitney if a significant difference was found with adjusted significance using Bonferroni correction. The secondary exploratory analysis used the CNSVS severity classification grade (described above and based on age-adjusted standardized scores) to compare the proportion of patients performing below average on each of the 8 cognitive domains using Fisher’s Exact Test to with odds ratio and associated 95% Confidence Intervals (CI) (24 total comparisons). The relationship between PROMs (specifically the DHI, THI, SSQ and ABC) and cognitive performance across 8 domains were compared using scatterplots for visualization and Pearson’s correlations. We had 24 correlations for DHI, SSQ12 and ABC (3 groups X 8 cognitive domains) and 16 correlations for THI (UVH group reported no tinnitus) for a total of 88 correlations.

## Results

A total number of 58 adults were recruited into the study (28 controls, 10 UHL, 10 MD, 10 UVH.) The age ranged from 25 to 82 years, with comparable mean age between all groups (*p* = 0.6). Additional demographic and clinical data of each group is depicted in Table 2. Etiology of UHL was predominately sudden idiopathic (n = 8), 1 had SNHL following stapes surgery and 1 had a cerebrospinal fluid leak that presented with SNHL. All patients with UVH had undergone vestibular rehabilitation therapy. All MD patients met criteria for definitive MD (29) (as described above), and were under either medical treatment or controlled their symptoms with lifestyle/dietary modifications. All had previously received intratympanic steroid injections, though none had gotten them on the day of cognitive testing. Reported prior vertigo episode ranged from 3 months to 12 months prior to testing. None of the MD patients had undergone surgical treatment for MD. One patient had probable vestibular migraine in addition to MD.

**Table 2:** Demographic table of participants divided by groups.

Group	Controls	Unilateral hearing loss (UHL)	Unilateral vestibular hypofunction (UVH)	Meniere’s Disease (MD)	*p*-value
*N*	28	10	10	10	
Age years, mean (SD) Range	59.14 (11.96) 41–78	55.3 (17.68) 25–76	58.5 (18.2) 24–82	52.7 (13.73) 32–78	*p* = 0.61
Sex *N* (%)	15 female (53.6%)	6 females (60%)	6 females (60%)	6 females (60%)	*p* = 0.985
PTA dB, mean (SD) Range: Better ear	16.97 (5.94), 6–26.25	15.88 (7.97), 2.5–25	16.25* (5.8), 10–25	11.00 (5.83), 3.75–22.5	*p* = 0.14
PTA dB, mean (SD) Range: Poorer ear	16.32 (6.3), 5–25	78.5 (19.42), 50–110	17.25* (5.7), 10–25	49.12 (16.57), 40–95	*p* < 0.001, normal hearing worse than MD (*p* = 0.003) and UHL (*p* < 0.001)
Duration of disease y, mean (SD) Range	NA	5.09 (6.1), 1–18	3.7 (3.2), 1–10	4.95 (3.3), 1–10	*p* = 0.4
Race^¥^, *N* (%):					*p* = 0.183
Caucasian	19 (67.9%)	4 (40%)	6 (60%)	9 (90%)	
Spanish/Hispanic	2 (7.1%)	2 (20%)	0 (0%)	2 (20%)	
Black or African American	3 (10.7%)	0 (0%)	2 (20%)	1 (10%)	
Asian American	2 (7.1%)	1 (10%)	1 (10%)	0 (0%)	
Asian / Pacific Islander	0 (0%)	3 (30%)	2 (20%)	0 (0%)	
Others	2 (7.1%)	0 (0%)	0 (0%)	0 (0%)	
Education Level, *N* (%)					*p* = 0.038, driven by graduate level education in the control group and missing data in the UVH group.
High school or equivalent	2 (7.1%)	2 (20%)	2 (20%)	2 (20%)	
Undergraduate (bachelor’s) degree	8 (28.6%)	5 (50%)	2 (20%)	3 (30%)	
Graduate/post-baccalaureate study	18 (64.3%)	2 (20%)	1 (10%)	3 (30%)	
Missing	0 (0%)	1 (10%)	5 (50%)	2 (20%)	
DHI, mean (SD) Range	0.36 (1.89), 0–10	21.8 (21.36), 0–54	34.8 (17.62), 8–62	40.2 (28.73), 0–78	*P* < 0.001, normal hearing sig lower than UHL (*p* = 0.021), UVH and MD (p < 0.001)
THI, mean (SD) Range	NA	33.6 (30.31), 0–92	4.2 (13.8), 0–42	35.4 (30.42), 0–82	*p* = 0.009, UVH sig lower than UHL *p* = 0.039, UVH sig lower than MD *p* = 0.016
SSQ12, mean (SD) Range	8.16 (1.15), 6.3–9.5	5.6 (1.49), 2.83–7.92	8.63 (1.17), 6–10	6.5 (2.2), 1.83–10	*P* < 0.001, UHL sig lower than normal hearing *p* = 0.022, UHL sig lower than UVH *p* = 0.001
ABC, mean (SD) Range	95.15% (5.88), 80–100	85.5% (10.6), 64–98	73.8% (23.17), 27–100	84.8% (17.13), 53–100	*P* = 0.003, normal hearing sig higher than UHL (*p* = 0.048) and UVH (*P* = 0.009)

ABC, Activities Balance Confidence Scale; DHI = the Dizziness Handicap Inventory questionnaire PTA = pure tone average; SSQ12, Speech, Spatial, and Qualities of Hearing Scale; THI, Tinnitus Handicap Inventory. All continuous variables were compared with independent samples Kruskal-Wallis test followed by Mann–Whitney is a significant difference was found with adjusted significance. All categorical variables were compared with Fisher’s Exact Test.

*Missing data for 5 patients.

^¥^ Totals > 10 because patients responded with more than one race.

**Figure 1:**
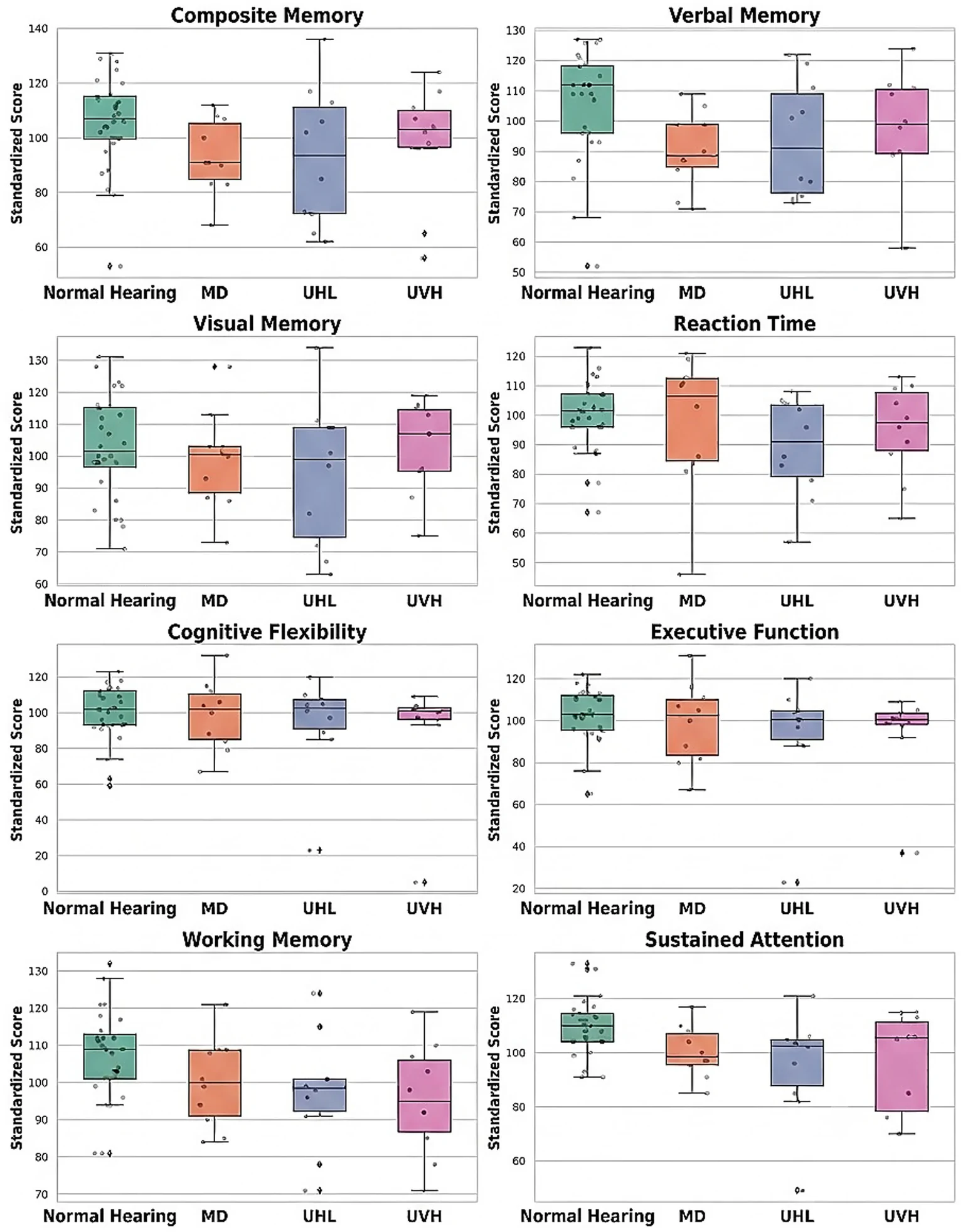
Descriptive statistics of all groups (controls, Unilateral Hearing Loss, UHL; Meniere’s Disease, MD; and Unilateral Vestibular Loss, UVL) per cognitive domain.

Table 3 and Figure 1 include descriptive statistics of all groups (control, UHL, MD and UVL) per cognitive domain. Most domains did not differ significantly between groups. Differences emerged in verbal memory (*H*^3^ = 9.28, *p* = 0.026), working memory (*H*^3^ = 7.77, *p* = 0.05) and sustained attention (*H*^3^ = 9.35, *p* = 0.03). After correcting for multiple comparisons, the MD group scored significantly lower than controls in verbal memory ( $p_{\text{Bonf}}=0.0174$ ). Additionally, the UHL group scored significantly lower than controls in sustained attention ( $p_{\text{Bonf}}=0.0391$ ).

**Table 3:** Descriptive statistics of cognitive outcomes across groups.

Cognitive Domain mean (SD), median (minimum, maximum)	Clinical group			
	Controls	MD	UHL	UVH
Composite Memory	105.54 (17.12), 107.0 (53, 131)	93.30 (13.63), 91.0 (68, 112)	93.10 (25.22), 93.5 (62, 136)	98.00 (21.59), 103.0 (56, 124)
Verbal Memory	106.11 (18.00), 112.0 (52, 127)	90.40 (12.69), 88.5 (71, 109)	93.90 (19.42), 91.0 (73, 122)	94.90 (22.15), 99.0 (58, 124)
Visual Memory	102.93 (15.98), 101.5 (71, 131)	98.70 (15.27), 100.5 (73, 128)	94.50 (22.88), 99.0 (63, 134)	103.00 (14.35), 107.0 (75, 119)
Reaction Time	99.96 (11.95), 101.5 (67, 123)	97.40 (23.24), 106.5 (46, 121)	89.00 (16.91), 91.0 (57, 108)	94.90 (15.72), 97.5 (65, 113)
Cognitive Flexibility	99.93 (15.63), 102.0 (59, 123)	98.70 (19.32), 102.0 (67, 132)	94.20 (26.98), 102.5 (23, 120)	91.10 (30.59), 101.0 (5, 109)
Executive Function	102.63 (12.69), 103.0 (65, 122)	98.70 (19.30), 102.5 (67, 131)	93.70 (26.56), 100.5 (23, 120)	94.70 (20.77), 100.5 (37, 109)
Working Memory	107.19 (12.15), 109.0 (81, 132)	100.00 (11.95), 100.0 (84, 121)	97.40 (15.50), 98.5 (71, 124)	95.50 (14.90), 95.0 (71, 119)
Sustained Attention	109.56 (10.23), 110.0 (91, 133)	100.40 (9.52), 98.5 (85, 117)	95.30 (19.65), 102.5 (49, 121)	96.10 (18.75), 105.5 (70, 115)

**Figure 2:**
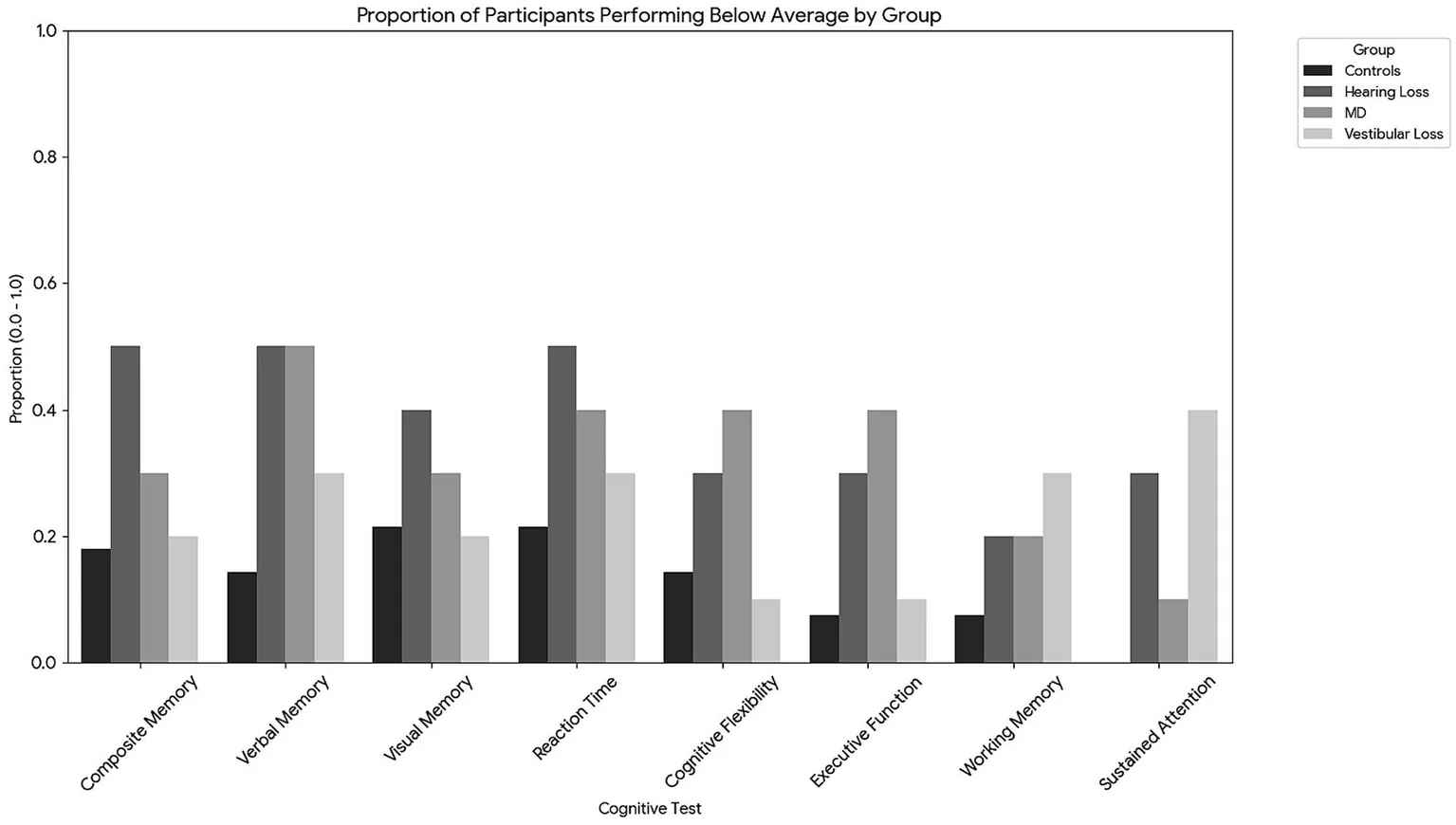
Proportion of participants performing below average (standardized score < 90) by clinical group (controls, Unilateral Hearing Loss, UHL; Meniere’s Disease, MD; and Unilateral Vestibular Loss, UVL) on each cognitive domain.

Figure 2 shows the proportion of individuals in each group performing “below average” (standardized score < 90) on each domain and the analysis appears in Table 4. There was a trend toward lower performance on the domains of verbal memory for UHL and MD groups, executive function for MD, and sustained attention for those with UHL or UVL. The latter 2 maintained significance after Bonferroni correction.

**Table 4:** Pairwise, two-tailed Fisher’s exact test comparisons for below-average (standardized score < 90) cognitive domain performance.

Cognitive domain	Comparison group	Below Ave/Total	Proportion (%)	Odds Ratio (95% CI)	Raw *p*-value	Bonferroni *p*-value
Composite Memory	Control	5/28	17.9%	–	–	–
	UHL vs. Control	5/10	50.0%	4.60 [0.95, 22.16]	0.0901	0.2703
	MD vs. Control	3/10	30.0%	1.97 [0.37, 10.40]	0.4112	1.0000
	UVH vs. Control	2/10	20.0%	1.15 [0.19, 7.14]	1.0000	1.0000
Verbal Memory	Control	4/28	14.3%	–	–	–
	UHL vs. Control	5/10	50.0%	6.00 [1.18, 30.63]	0.0362	0.1086
	MD vs. Control	5/10	50.0%	6.00 [1.18, 30.63]	0.0362	0.1086
	UVH vs. Control	3/10	30.0%	2.57 [0.46, 14.32]	0.3510	1.0000
Visual Memory	Control	6/28	21.4%	–	–	–
	UHL vs. Control	4/10	40.0%	2.44 [0.52, 11.57]	0.4036	1.0000
	MD vs. Control	3/10	30.0%	1.57 [0.31, 7.99]	0.6731	1.0000
	UVH vs. Control	2/10	20.0%	0.92 [0.15, 5.51]	1.0000	1.0000
Reaction Time	Control	6/28	21.4%	–	–	–
	UHL vs. Control	5/10	50.0%	3.67 [0.79, 16.99]	0.1161	0.3483
	MD vs. Control	4/10	40.0%	2.44 [0.52, 11.57]	0.4036	1.0000
	UVH vs. Control	3/10	30.0%	1.57 [0.31, 7.99]	0.6731	1.0000
Cognitive Flexibility	Control	4/28	14.3%	–	–	–
	UHL vs. Control	3/10	30.0%	2.57 [0.46, 14.32]	0.3510	1.0000
	MD vs. Control	4/10	40.0%	4.00 [0.77, 20.82]	0.1701	0.5103
	UVH vs. Control	1/10	10.0%	0.67 [0.07, 6.79]	1.0000	1.0000
Executive Function	Control	2/27	7.4%	–	–	–
	UHL vs. Control	3/10	30.0%	5.36 [0.74, 38.64]	0.1102	0.3306
	MD vs. Control	4/10	40.0%	8.33 [1.23, 56.67]	0.0347	0.1041
	UVH vs. Control	1/10	10.0%	1.39 [0.11, 17.24]	1.0000	1.0000
Working Memory	Control	2/27	7.4%	–	–	–
	UHL vs. Control	2/10	20.0%	3.12 [0.38, 25.92]	0.2914	0.8742
	MD vs. Control	2/10	20.0%	3.12 [0.38, 25.92]	0.2914	0.8742
	UVH vs. Control	3/10	30.0%	5.36 [0.74, 38.64]	0.1102	0.3306
Sustained Attention	Control	0/27	0.0%	–	–	–
	UHL vs. Control	3/10	30.0%	NA*	**0.0154**	**0.0462**
	MD vs. Control	1/10	10.0%	NA	0.2703	0.8109
	UVH vs. Control	4/10	40.0%	NA	**0.0032**	**0.0096**

Each clinical cohort is compared back to the control group. The odds ratio (OR) with its corresponding 95% confidence interval (CI), raw *p*-value, and Bonferroni-corrected *p*-value are reported for each pairwise comparison.

*An Odds Ratio was not calculated for sustained attention because the control group had zero (0%) individuals performing below average. Bold values are MD, Menieres Disease; UHL, unilateral hearing loss; UVH, unilateral vestibular loss.

**Figure 3:**
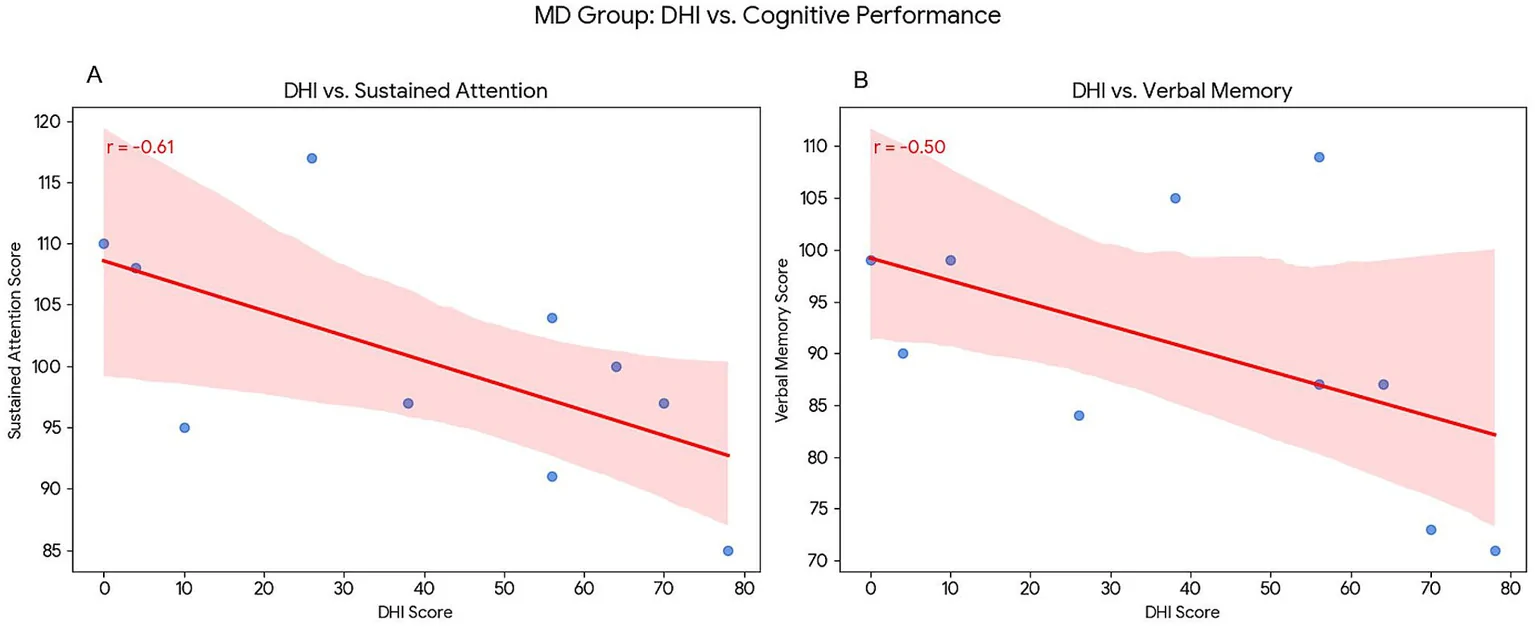
**(A,B)** Meniere’s Disease (MD) group: Correlation between DHI (Dizziness Handicap Index score) and cognitive performance by domain: **(A)** Sustained attention; **(B)** Verbal memory.

**Figure 4:**
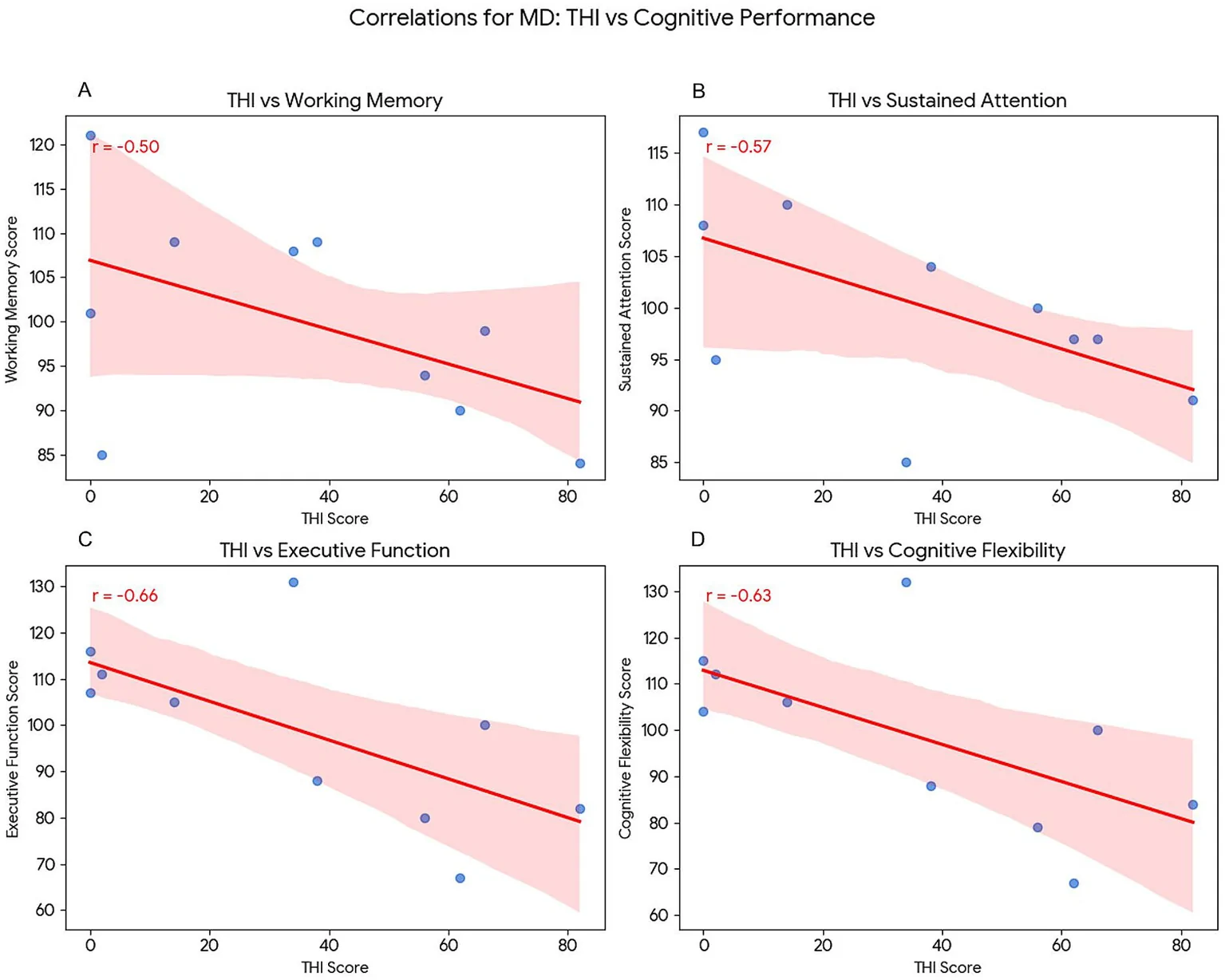
**(A–D)** Meniere’s Disease (MD) group: Correlation between THI (Tinnitus Handicap Index) and cognitive performance by domain: **(A)** working memory; **(B)** sustained attention; **(C)** executive function; **(D)** cognitive flexibility.

**Figure 5:**
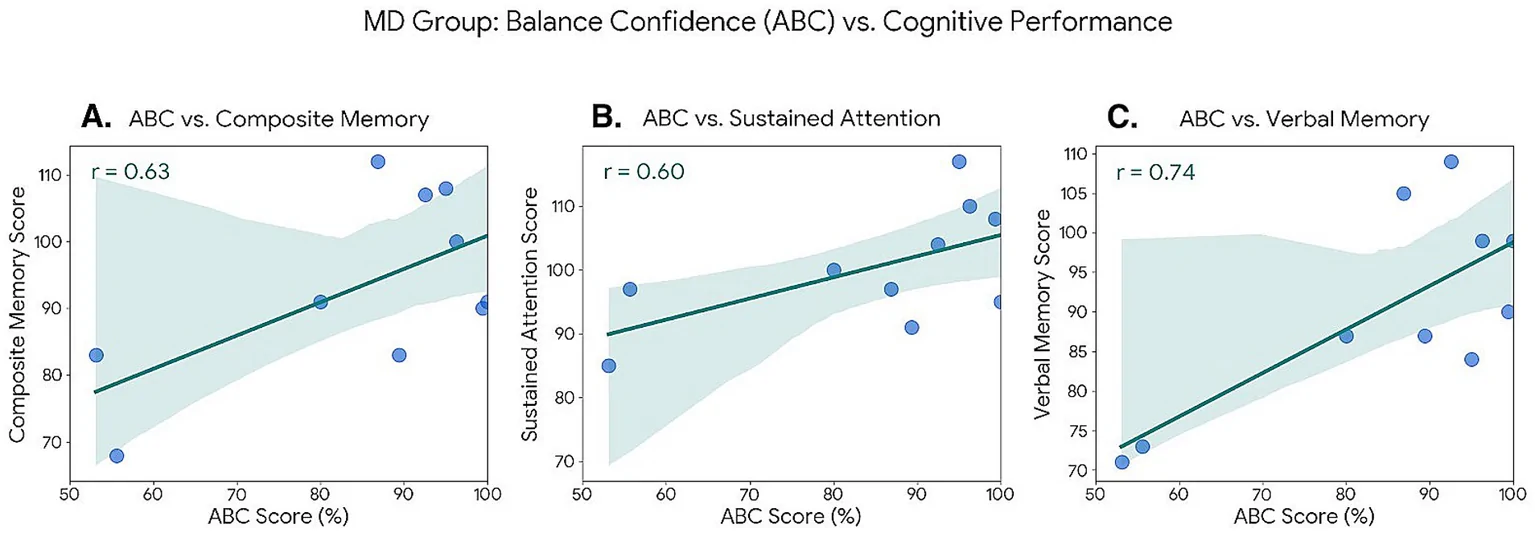
**(A–C)** Meniere’s Disease (MD) group: Correlation between balance confidence (Activities of Balance Confidence, ABR) and cognitive performance by domain: **(A)** composite memory; **(B)** sustained attention; **(C)** verbal memory.

Correlations between patient-reported outcomes and cognitive domains with respective 95% CI appear in Supplementary Tables 1–4 (DHI, THI, SSQ12 and ABC). There were a few notable trends [e.g., at least moderate correlations (0.5 or above) between PROM and cognitive domains (Figures 3–5)], especially for the MD group.

Among patients with MD: Lower DHI scores (less disability due to dizziness) were associated with better sustained attention (Figure 3A, *R* = −0.61) and verbal memory (Figure 3B, *R* = −0.5). Higher SSQ12 (reflecting better perceived hearing function) was associated with better verbal memory (*R* = 0.58). Lower THI scores (less tinnitus) were associated with better working memory (Figure 4A, *R* = −0.50), sustained attention (Figure 4B, *R* = −0.57), executive function (Figure 4C, R = -0.66) and cognitive flexibility (Figure 4D, *R* = −0.63). Higher ABC scores were associated with better composite memory (Figure 5A, *R* = 0.63), better sustained attention (Figure 5B, *R* = 0.596), and better verbal memory (Figure 5C, *R* = 0.74).

**Figure 6:**
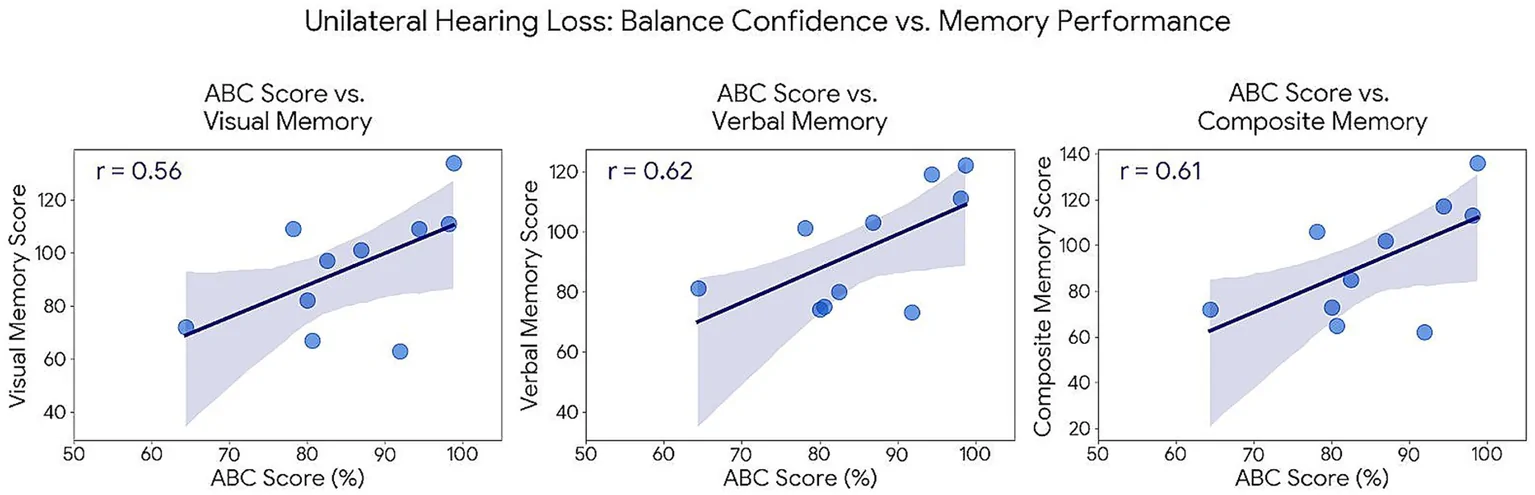
Unilateral hearing loss (UHL) group: correlation between cognitive scores and balance confidence (Activities of Balance Confidence, ABC).

Among patients with UHL: Lower DHI scores were associated with better working memory (*R* = −0.5). Higher ABC scores (Figures 6A–C) were associated with better visual memory (*R* = 0.56), verbal memory (*R* = 0.62) and composite memory (*R* = 0.61).

No important correlations were observed for UVH. Overall, if patients had no tinnitus they were included in the analysis as THI = 0, however, because only 1 patient with UVH reported experiencing tinnitus, correlations with THI could not be obtained for this group.

## Discussion

This preliminary investigation of cognitive function in unilateral audiovestibular disorders using a standardized, comprehensive, non-auditory based test battery covering 8 domains showed several notable trends. First, some differences in cognitive performance may exist between groups, and these differences localize along specific domains. Second, several moderate correlations between cognitive function and symptom severity, especially among those with MD, as measured by standardized PROMs, suggest an association between tinnitus, dizziness and cognitive function for those burdened with severe dysfunction. This causal hypothesis needs to be tested in future studies.

There has been limited prior investigation of cognitive function among adults with acquired UHL. Our preliminary investigation suggests that the cognitive domains of memory and sustained attention may be relevant to those with UHL, and supports work of prior studies. Individuals with UHL must allocate additional mental resources to overcome the lack of binaural input, and this listening effort may impact cognitive function. Su et al. utilized pupillometry to assess listening effort in patients with a profound UHL (i.e., single-sided deafness; SSD) during verbal tests of working memory and found pupillary behavior indicative of higher attentional demands, greater cognitive resource allocation, fatigue and cognitive strain (13). Cognitive performance may also reflect cortical changes that occur after sudden or acquired hearing loss (36). Alterations in both auditory and non-auditory brain regions following sudden unilateral sensorineural hearing loss (SSNHL) have been discovered using resting-state fMRI, with recent data suggesting SSNHL may lead to broader alterations in networks involved in attention, memory, and executive function (37). Further study of the cognitive burden of acquired UHL is warranted as it has the potential to inform treatment decisions, especially in older patients or those who may have other risk factors for cognitive impairment. Unlike bilateral hearing loss, it is currently unknown whether treatment of UHL can impact cognition. Currently, clinical decision-making for UHL is complex due to a wide range of treatment options, including conservative observation, hearing aid amplification, as well as surgical and non-surgical contralateral routing of sound (CROS) options and cochlear implantation (38–40). Our pilot data identified cognitive domains that, if confirmed in future larger studies, may be targeted by future intervention studies to better understand both the burden of UHL as well as the potential impact of various treatment options.

Individuals with unilateral MD also experience ipsilateral hearing loss, and in the present pilot, both groups differed from controls in the domain of verbal memory. MD patients also demonstrated a relationship between symptom severity and cognitive performance, with less tinnitus and dizziness and higher balance confidence associated with better cognitive performance across multiple domains. In general, and certainly in a small pilot, it is important to acknowledge the inherent heterogeneity of MD patients (41). Even among patients meeting criteria for definitive MD, there can be significant variability between patients in clinical presentation, symptomatology and treatment response. Both the current pilot and a prior study on MD by Zhong et al. (24) found a relationship between symptom severity and cognitive performance, with those experiencing greater dizziness having worse cognitive scores. Using the Montreal Cognitive Assessment (MoCA) and the DHI, Zhong et al. (24) compared healthy controls to MD patients before and after treatment. At baseline, controls and MD patients differed on the MoCA only in the subdomain of memory, without significant differences in other subdomain scores. This is similar to the present pilot which suggested differences between controls and MD patients on tests of composite and verbal memory, as well as sustained attention. Unlike global tests of cognition, domain-based testing may be more useful in audiovestibular diseases with a high degree of clinical heterogeneity such as MD, where testing may identify patients at higher risk of cognitive dysfunction. Additionally, given the known confounder of hearing loss among MD patients, cognitive testing that does not rely on auditory stimuli is preferred. Other non-auditory assessments such as the Cognitive Failures Questionnaire (CFQ) which have shown sensitivity to cognitive dysfunction a variety of vestibular disorders have had limited success in MD, highlighting potential value of domain-based testing in the MD population (22, 42, 43).

The present pilot study also pointed to a possible correlation between tinnitus severity and cognitive performance in MD, an area not previously explored. Among the MD group, less tinnitus was associated with better sustained attention, memory, executive function and cognitive flexibility. Outside of MD, tinnitus has been associated with attentional deficits, poorer executive function, processing speed, worse short-term memory, and increased cognitive load retrieval (27, 44). A negative correlation between THI scores and internetwork connectivity between the dorsal attentional and auditory networks has been found on fMRI, suggesting a potentially broad influence of tinnitus severity across multiple auditory and non-auditory regions (37). Tinnitus is a key feature of MD and those with high symptom burden may warrant cognitive assessment and treatment specifically targeting tinnitus symptoms. Tinnitus retraining therapy has been shown to enhance cognitive processing in a limited study on individuals with tinnitus and normal hearing, signifying an area ripe for future research (45). For those with tinnitus and non-serviceable UHL, unilateral cochlear implantation has shown remarkable efficacy in reducing tinnitus symptoms (11, 46).

Prior studies have investigated cognitive function in unilateral and bilateral chronic vestibulopathy, consistently finding greater impairment among those with bilateral disease (18). Popp et al. (47) found patients with unilateral patients were impaired in visuospatial and processing speed. Specific cognitive tests employed by Popp et al. and the current pilot differed, with the exception of the Stroop task (Table 1) in which the results were similar (neither study found a difference between UVH and controls) (47). However, there is significant conceptual overlap between the domain of processing speed [Popp et al. (47)] and sustained attention (present study), both of which were impaired in patients UVH. The relationship between symptom severity and cognition in this population remains poorly understood. Ahmad et al. (48) investigated symptom severity and cognition in uni- and bilateral vestibulopathy using domain-based testing and found limited evidence of cognitive dysfunction in the UVH group [only sustained attention in the present study; visuospatial in Ahmad et al. (48)] and no relationship with symptom severity. It is possible that the results reflect the effects of prior treatment: in both studies, patients with UVH had undergone vestibular rehabilitation. Future research on UVH that evaluates cognition pre- and post-treatment is warranted.

Finally, while evaluating dementia risk was beyond the scope of this pilot study, understanding the potential association between unilateral audiovestibular disease and cognitive decline remains a critical research gap. As reviewed above, current understanding of hearing and vestibular dysfunction as a modifiable risk factor for cognitive decline and dementia had been nearly completely reliant on data in bilateral disease. However, recently, UHL was recently linked to dementia in a large epidemiologic study of the UK Biobank (3). Similarly, unilateral vestibular dysfunction was also associated with non-spatial cognitive abilities and dementia risk in a prospective study (20).

There were multiple limitations to the current study. First, this was a pilot study thus the sample size was based on feasibility and designed to generating preliminary effect-size estimates for future, adequately-powered studies. Thus, with only 10 participants in each clinical group, estimates are likely to be unstable, particularly given the number of comparisons and correlations performed. Likewise, we focused on the estimates of the correlations rather than their statistical significance which is heavily influenced by sample size. Though clinical groups were clearly defined, we had limited ability to control for all the many disease and demographic confounders related to cognitive function. While the groups did not differ in age or other demographic variables, controls did achieve a higher level of education (Table 2) which may influence their cognitive performance and their generalizability to the general population. In the UHL group, a few patients had higher than expected scores on the DHI, despite denying dizziness to their provider. While none had episodic vertigo or met MD criteria, it is possible that some UHL patients represent an evolving or early form of Meniere’s disease that has not yet met clinical MD criteria. Thus, replication of the current paradigm in larger populations is necessary to confirm these findings. The test battery did not include clinical screening tests of cognitive function, such as the Montreal Cognitive Assessment or the Mini-mental status exam. CNSVS is a cognitive assessment tool, but not a substitute for a comprehensive neuropsychological evaluation. While patients with known neurocognitive disease were excluded and the average age of study participants suggests risk of undiagnosed dementia or age-related cognitive decline would be low, it is possible that this study included patients with global cognitive impairment.

The present study explored domain-specific differences in cognitive performance between controls and patients with unilateral audiovestibular disease, providing preliminary data for future studies that further explore this relationship. Additionally, this pilot study suggests relevant domains that may be considered in future studies evaluating cognitive performance after available treatments, such as hearing aids, cochlear implants, tinnitus retraining therapy and vestibular rehabilitation, as well as medication or surgical interventions.

## Conclusion

Cognitive effects of unilateral audiovestibular disorders represent an important knowledge gap in our understanding of these disorders. In this exploratory pilot study, UHL, MD and UVH demonstrated differences in cognitive performance compared to controls along domains related to memory and sustained attention that may reflect challenges unique to hearing loss, vestibular loss or a combination. Severity of disease related symptoms, specifically dizziness and tinnitus, may also relate to cognitive performance, especially in patients with MD. Further study exploring the cognitive burden of unilateral audiovestibular disease is warranted. Future studies have the potential to inform treatment decisions, especially in older patients or those who may have other risk factors for cognitive impairment.

## Data Availability

The raw data supporting the conclusions of this article will be made available by the authors, without undue reservation.
