# Supplementary material for: Cognitive performance, dizziness and tinnitus among adults with unilateral audiovestibular disorders
Source: Front Neurol. 2026 Aug 17;17:1911120. doi: 10.3389/fneur.2026.1911120 (PMC13526580; doi:10.3389/fneur.2026.1911120)
Supplement: Supplementary file 1 [file Table_1.DOCX]

**Supplementary Table 1. Pearson correlations between DHI score and cognitive domains across clinical groups**

| **Cognitive Domain** | **MD (n = 10)** | | | **UHL (n = 10)** | | | **UVH (n = 10)** | | |
| --- | --- | --- | --- | --- | --- | --- | --- | --- | --- |
|  | **r** | **p-value** | **95% CI** | **r** | **p-value** | **95% CI** | **r** | **p-value** | **95% CI** |
| **Composite Memory** | -0.418 | 0.229 | [-0.83, 0.29] | -0.241 | 0.503 | [-0.76, 0.46] | 0.314 | 0.377 | [-0.39, 0.79] |
| **Verbal Memory** | -0.495 | 0.146 | [-0.86, 0.20] | -0.278 | 0.437 | [-0.77, 0.43] | 0.242 | 0.500 | [-0.46, 0.76] |
| **Visual Memory** | -0.174 | 0.631 | [-0.72, 0.51] | -0.172 | 0.635 | [-0.72, 0.51] | 0.406 | 0.244 | [-0.30, 0.83] |
| **Reaction Time** | -0.129 | 0.723 | [-0.70, 0.55] | -0.002 | 0.995 | [-0.63, 0.63] | -0.094 | 0.797 | [-0.68, 0.57] |
| **Cognitive Flexibility** | -0.124 | 0.732 | [-0.70, 0.55] | -0.315 | 0.375 | [-0.79, 0.39] | 0.030 | 0.935 | [-0.61, 0.65] |
| **Executive Function** | -0.144 | 0.691 | [-0.71, 0.53] | -0.286 | 0.423 | [-0.78, 0.42] | 0.052 | 0.886 | [-0.60, 0.66] |
| **Working Memory** | -0.065 | 0.858 | [-0.67, 0.59] | -0.520 | 0.124 | [-0.87, 0.16] | 0.276 | 0.440 | [-0.43, 0.77] |
| **Sustained Attention** | -0.614 | **0.059** | [-0.90, 0.03] | -0.258 | 0.472 | [-0.76, 0.44] | 0.432 | 0.213 | [-0.27, 0.83] |
