# Supplementary material for: Cognitive performance, dizziness and tinnitus among adults with unilateral audiovestibular disorders
Source: Front Neurol. 2026 Aug 17;17:1911120. doi: 10.3389/fneur.2026.1911120 (PMC13526580; doi:10.3389/fneur.2026.1911120)
Supplement: Supplementary file 2 [file Table_2.DOCX]

**Supplementary Table 2. Pearson correlations between THI total score and cognitive domains across clinical groups**

| **Cognitive Domain** | **MD (n = 10)** | | | **UHL (n = 10)** | | |
| --- | --- | --- | --- | --- | --- | --- |
|  | **r** | **p-value** | **95% CI** | **r** | **p-value** | **95% CI** |
| **Composite Memory** | -0.328 | 0.355 | [-0.79, 0.38] | 0.067 | 0.854 | [-0.59, 0.67] |
| **Verbal Memory** | -0.152 | 0.675 | [-0.71, 0.53] | 0.126 | 0.729 | [-0.55, 0.70] |
| **Visual Memory** | -0.349 | 0.323 | [-0.80, 0.36] | 0.035 | 0.924 | [-0.61, 0.65] |
| **Reaction Time** | -0.395 | 0.259 | [-0.82, 0.31] | -0.391 | 0.264 | [-0.82, 0.32] |
| **Cognitive Flexibility** | -0.630 | 0.051 | [-0.90, -0.00] | -0.176 | 0.626 | [-0.73, 0.51] |
| **Executive Function** | **-0.661** | **0.037** | [-0.91, -0.05] | -0.181 | 0.617 | [-0.73, 0.51] |
| **Working Memory** | -0.496 | 0.145 | [-0.86, 0.19] | -0.028 | 0.939 | [-0.65, 0.61] |
| **Sustained Attention** | -0.572 | 0.084 | [-0.88, 0.09] | 0.179 | 0.621 | [-0.51, 0.73] |

*** Tinnitus correlations were not calculable for the UVH cohort due to an invariant floor effect (THI score of 0 across patients).
