# Supplementary material for: Cognitive performance, dizziness and tinnitus among adults with unilateral audiovestibular disorders
Source: Front Neurol. 2026 Aug 17;17:1911120. doi: 10.3389/fneur.2026.1911120 (PMC13526580; doi:10.3389/fneur.2026.1911120)
Supplement: Supplementary file 3 [file Table_3.DOCX]

**Supplementary Table 3. Summary of Pearson correlations between total SSQ12 scores and cognitive domains**

| **Cognitive Domain** | **MD Group (n = 10)** | | | **UHL Group (n = 10)** | | | **UVH Group (n = 10)** | | |
| --- | --- | --- | --- | --- | --- | --- | --- | --- | --- |
|  | **r** | **p-value** | **95% CI** | **r** | **p-value** | **95% CI** | **r** | **p-value** | **95% CI** |
| **Composite Memory** | 0.297 | 0.404 | [-0.41, 0.78] | -0.309 | 0.386 | [-0.79, 0.40] | -0.067 | 0.855 | [-0.67, 0.59] |
| **Verbal Memory** | 0.582 | *0.078* | [-0.08, 0.89] | -0.296 | 0.406 | [-0.78, 0.41] | -0.105 | 0.773 | [-0.69, 0.56] |
| **Visual Memory** | -0.009 | 0.980 | [-0.64, 0.62] | -0.335 | 0.345 | [-0.80, 0.37] | -0.002 | 0.996 | [-0.63, 0.63] |
| **Reaction Time** | -0.311 | 0.381 | [-0.79, 0.40] | 0.412 | 0.237 | [-0.29, 0.83] | 0.158 | 0.663 | [-0.52, 0.72] |
| **Cognitive Flexibility** | -0.010 | 0.979 | [-0.64, 0.62] | 0.391 | 0.264 | [-0.32, 0.82] | -0.083 | 0.819 | [-0.68, 0.58] |
| **Executive Function** | -0.014 | 0.969 | [-0.64, 0.62] | 0.378 | 0.281 | [-0.33, 0.81] | -0.118 | 0.745 | [-0.70, 0.55] |
| **Working Memory** | -0.358 | 0.309 | [-0.81, 0.35] | 0.142 | 0.695 | [-0.54, 0.71] | 0.088 | 0.808 | [-0.57, 0.68] |
| **Sustained Attention** | -0.017 | 0.963 | [-0.64, 0.62] | 0.077 | 0.832 | [-0.58, 0.67] | -0.014 | 0.970 | [-0.64, 0.62] |
