# Supplementary material for: Cognitive performance, dizziness and tinnitus among adults with unilateral audiovestibular disorders
Source: Front Neurol. 2026 Aug 17;17:1911120. doi: 10.3389/fneur.2026.1911120 (PMC13526580; doi:10.3389/fneur.2026.1911120)
Supplement: Supplementary file 4 [file Table_4.DOCX]

**Supplementary Table 4. Pearson correlations between ABC score and cognitive domains across clinical groups**

| **Cognitive Domain** | **MD Group (n = 10)** | | | **UHL Group (n = 10)** | | | **UVH Group (n = 10)** | | |
| --- | --- | --- | --- | --- | --- | --- | --- | --- | --- |
|  | **r** | **p-value** | **95% CI** | **r** | **p-value** | **95% CI** | **r** | **p-value** | **95% CI** |
| **Composite Memory** | 0.626 | *0.053* | [-0.01, 0.90] | 0.608 | *0.062* | [-0.04, 0.90] | -0.121 | 0.739 | [-0.70, 0.55] |
| **Verbal Memory** | **0.743** | **0.014** | [0.21, 0.94] | 0.620 | *0.056* | [-0.02, 0.90] | -0.122 | 0.738 | [-0.70, 0.55] |
| **Visual Memory** | 0.287 | 0.421 | [-0.42, 0.78] | 0.562 | *0.091* | [-0.11, 0.88] | -0.137 | 0.706 | [-0.71, 0.54] |
| **Reaction Time** | -0.080 | 0.826 | [-0.68, 0.58] | 0.075 | 0.836 | [-0.58, 0.67] | -0.020 | 0.956 | [-0.64, 0.62] |
| **Cognitive Flexibility** | -0.230 | 0.523 | [-0.75, 0.47] | 0.036 | 0.921 | [-0.61, 0.65] | 0.102 | 0.779 | [-0.56, 0.69] |
| **Executive Function** | -0.215 | 0.551 | [-0.74, 0.48] | 0.057 | 0.876 | [-0.59, 0.66] | 0.068 | 0.852 | [-0.59, 0.67] |
| **Working Memory** | -0.063 | 0.863 | [-0.67, 0.59] | 0.429 | 0.216 | [-0.27, 0.83] | -0.126 | 0.729 | [-0.70, 0.55] |
| **Sustained Attention** | 0.596 | *0.069* | [-0.05, 0.89] | 0.438 | 0.206 | [-0.27, 0.84] | -0.252 | 0.483 | [-0.76, 0.45] |
